# Gustaf Von Düben’s Treatise on Microscopical Diagnosis

**Published:** 1860-03

**Authors:** 


Art. IV.—*Gustaf Von Düben's Treatise on Microscopical Diagnosis.* With 71 Engravings. Translated, with additions, by Louis Bauer, M.D., M.R.C.S., Eng., etc. 8vo., pp. 82. New York: John Wiley, 1859.

As a means of anatomical research, assisting the eye in the investigation of form and structure, the microscope has effected a very important work in the advancement of medical science within the past twenty-five years. It has, for instance, by disclosing the intimate construction of the several tissues, and of different products, such as pus and lymph, both enabled and obliged authors to employ greater precision of language than they did before they possessed this knowledge. It has exhibited to us the material agents by which most of the functions of life are carried on, and has thus brought us at least a step toward the comprehension of those actions themselves.

The early advocates of microscopy were led by their enthusiasm to claim far too much for their favorite instrument; they thought it would supersede in a great degree other means of diagnosis, and in their haste they took ground which by riper experience was shown to be untenable. An experienced microscopist becomes acquainted, in gaining his experience, with the various forms which are brought under his notice, and learns to distinguish them from one another. An inexperienced microscopist can only hope to acquire diagnostic skill by the same accumulative process. Hence we think that the title of the work before us could only be properly applied to a comprehensive survey of the minute anatomy of the human body and its parasites, in health as well as in disease. The mere use of the microscope in diagnosis, we take it, would be too narrow a subject to form the basis of a treatise.

But even in this little volume of seventy-six pages, the offspring of the labors of Von Düben, Tutschek, and Bauer, many matters are introduced which are far from relevant to its alleged purpose; others, of great importance to the reader, are omitted. Thus we are told (p. 32) that:—

"In reference to microscopical diagnosis, milk may become the object of interest in two ways.

"1. The relative proportions of the milk-constituents.

"Since Donné, Zettwach, and Girard have ascribed to the collostrum* corpuscles in the milk obnoxious effects upon the suckling, they have been the subject of close examination. It should, however, be borne in mind, that a few collostrum corpuscles physiologically occur in the milk for some time after childbirth, without at all vitiating its nutritious or wholesome character. But a large number of those elements would be evidence of congestion or inflammation of the mammary gland." (Even had this been proved, as it has not to our knowledge, we think that such evidence of these disorders would be altogether unnecessary, in the presence of much more important signs.) "In some diseases of nursing women, the collostrum corpuscles generate in great abundance. Thus in rheumatism, exanthemata, and typhus lactantium. In the last, according to Lehmann, are occasionally found regular exudation corpuscles, with traces of fat granules.

"The presence of collostrum corpuscles in large numbers is important evidence of recent parturition, and therefore of great moment in a forensic point of view.

"2. Abnormal substances in the milk.

"It is said that after contusions of the mammary gland the milk shows red blood corpuscles and fragments of blood-clots and pus cells in incipient mammary abscess. We have never observed those elements, whereas we have met in the latter instance with collostrum corpuscles four months after confinement. Nor have we any positive idea as to the obnoxious results of those abnormal elements upon the infant. Their observation may, however, assist in forming an early diagnosis of incipient abscess.

"In Wagner's Encyclopedia of Physiology we read, vol. i., page 470, 'Sometimes the milk, after having been exposed to the action of atmospheric air, assumes a *blue*, but rarely a yellow color, beginning at the surface and gradually extending over the whole liquid. When examined by J. Fuchs, he found infusoria to be the cause of discoloration; vibrio cyanogenus being in the blue milk and vibrio xantogenus in the yellow. The infusoria themselves are stated to be colorless, but they possess the quality of discoloring milk.'

---

* The spelling and punctuation are as in the original.

"In red milk C. Nægeli has observed Protococcus. As far as known cancer cells have as yet not been found in the milk; whereas mammary cancer occasionally gives rise to the secretion of normal milk and collostrum. In a cancerous tumor of the breast, removed by Dr. Svalin, the Author had an opportunity of confirming the last-mentioned fact in addition to the development of milk secretion as demonstrated by Lammerts Van Bueren."

The vague and unpractical character of these remarks can hardly require comment; they afford a fair sample of the rest of the volume. Urinary sediments, the microscopical diagnosis of which is really important, are disposed of in six pages and a half. Dr. Bauer promises in his preface to "evade" the use of technical terms; yet we find "sporotrych-development," "lumen," "neoplastic," "urate-infarcts," and other equally formidable expressions, introduced as if they were household words.

Those of the profession who desire to use the microscope, and, as we have already remarked, its utility as a means of research is very great, are much in want of a volume in which they may find the information necessary to them as beginners. Many failures and a long course of wearisome effort must precede their attainment of an available degree of facility even in the manipulation of the instrument and its accessories. Much of this might be avoided were a book at hand which should give suitable instructions for beginners. Such a work should be more exclusively medical than the admirable treatise of Dr. Carpenter, less costly than that of Quekett, more comprehensive than that of Robin, and more practical than that of Beale. The Micrographic Dictionary, valuable as it is, cannot be generally possessed on account of its high price, and its contents are of necessity arranged in a way unsuitable for a manual. The production of such a book would require much labor, since many parts of the subject would have to be condensed as much as possible; but it would render an essential service to medical science, and the profession would doubtless extend to it a practical welcome.

